# A Josephson junction based on a highly disordered superconductor/low-resistivity normal metal bilayer

**DOI:** 10.3762/bjnano.11.71

**Published:** 2020-06-02

**Authors:** Pavel M Marychev, Denis Yu Vodolazov

**Affiliations:** 1 Institute for Physics of Microstructures, Russian Academy of Sciences, Nizhny Novgorod, 603950, Russia

**Keywords:** normal metal–superconductor bilayer, Josephson junction, Joule heating

## Abstract

We calculate the current–phase relation (CPR) of a SN-S-SN Josephson junction based on a SN bilayer of variable thickness composed of a highly disordered superconductor (S) and a low-resistivity normal metal (N) with proximity-induced superconductivity. In such a junction, the N layer provides both a large concentration of phase in the weak link and good heat dissipation. We find that when the thickness of the S and the N layer and the length of the S constriction are about the superconducting coherence length the CPR is single-valued and can be close to a sinusoidal shape. The product *I*_c_*R*_n_ can reach Δ(0)/2|e| (*I*_c_ is the critical current of the junction, *R*_n_ is its normal-state resistance, Δ(0) is the superconductor gap of a single S layer at zero temperature). Our calculations show, that the proper choice of the thickness of the N layer leads both to nonhysteretic current–voltage characteristics even at low temperatures and a relatively large product *I*_c_*R*_n_.

## Introduction

Josephson junctions are of interest for applications such as voltage standards [1], SQUID magnetometers [2], particle detectors [3], and energy-efficient superconductor logic and memory circuits [4–5]. These applications need to have a large critical current *I*_c_ to achieve high noise immunity. Also many of these applications require to have a nonhysteretic current–voltage characteristic (IVC) and a large characteristic voltage *V*_c_ = *I*_c_*R*_n_, where *R*_n_ is the normal-state resistance of the junction.

Tunnel superconductor–insulator–superconductor (SIS) Josephson junctions are characterized by small critical current densities (significantly smaller than the depairing current density of superconducting electrodes) and a hysteretic IVC (the latter is related with the large capacitance of the insulator layer), which restricts their applicability. Elimination of hysteresis in SIS junctions requires an external resistor or a more complex circuitry. S-c-S Josephson junctions (where “c” is a geometric constriction) have a small capacitance of the weak link and a high critical current (about the magnitude of the depairing current of a superconductor), which allows one to obtain high noise immunity. But due to large critical current and bad heat dissipation their IVCs are hysteretic due to Joule heating (
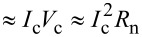
) and the subsequent formation of a stable region with suppressed superconductivity (a so-called “hot spot”) at *I > I*_c_ [6–9]. At temperatures near the critical temperature *T*_c_ the hysteresis is absent because of the low *I*_c_ and, therefore, small dissipation, but this leads to a small voltage *V*_c_.

Therefore, eliminating the thermal hysteresis without sacrificing the voltage *V*_c_ is important, albeit a nontrivial problem. One solution is a normal metal shunt either on top of the junction [10] or at a distance from it [11–12]. The resistance and the position of the shunt play an important role and they can lead to a reduction of the junction characteristics because of the proximity effect or a very small shunt resistance. In [13–14], it was proposed to use a variable-thickness SN-N-SN bilayer in which the superconducting layer is partially (or entirely) etched by means of a focused ion beam. A sufficiently thick normal metal layer act as a good thermal bath, which yields a nonhysteretic current–voltage characteristic even at low temperatures. However, the increase of the thickness of the N layer leads to a significant decrease of *R*_n_ and, hence, to smaller values of *V*_c_.

In our work, we calculate the current–phase relation and heating effects in SN-S-SN Josephson junctions of variable thickness based on a thin dirty superconductor with large normal-state resistivity, ρ_S_ ≥ 100 μΩ·cm, and a thin normal metal layer with low resistivity, ρ_N_ ≥ 2 μΩ·cm. In such a thin SN bilayer the superconducting current mainly flows in the N layer (due to proximity-induced superconductivity and ρ_S_/ρ_N_ ≫ 1), and the critical current of the SN bilayer may exceed the critical current of a single S layer if the thickness of the S and the N layers are about the superconducting coherence length [15]. Because of the large diffusion coefficient, *D*_N_ ≫ *D*_S_, the N layer provides both a large phase concentration in the constriction leading to a single-valued current–phase relation (CPR) and an effective thermal bath into which the heat from the junction area could be dissipated, resulting in nonhysteretic IVC even at relatively low temperatures. We also find that in comparison with a SN-N-SN junction, the critical current density could be similar to the depairing current density of the S layer, which makes it possible to obtain *I*_c_*R*_n_ ≈ Δ(0)/2|e|.

## Model

**Figure 1:**
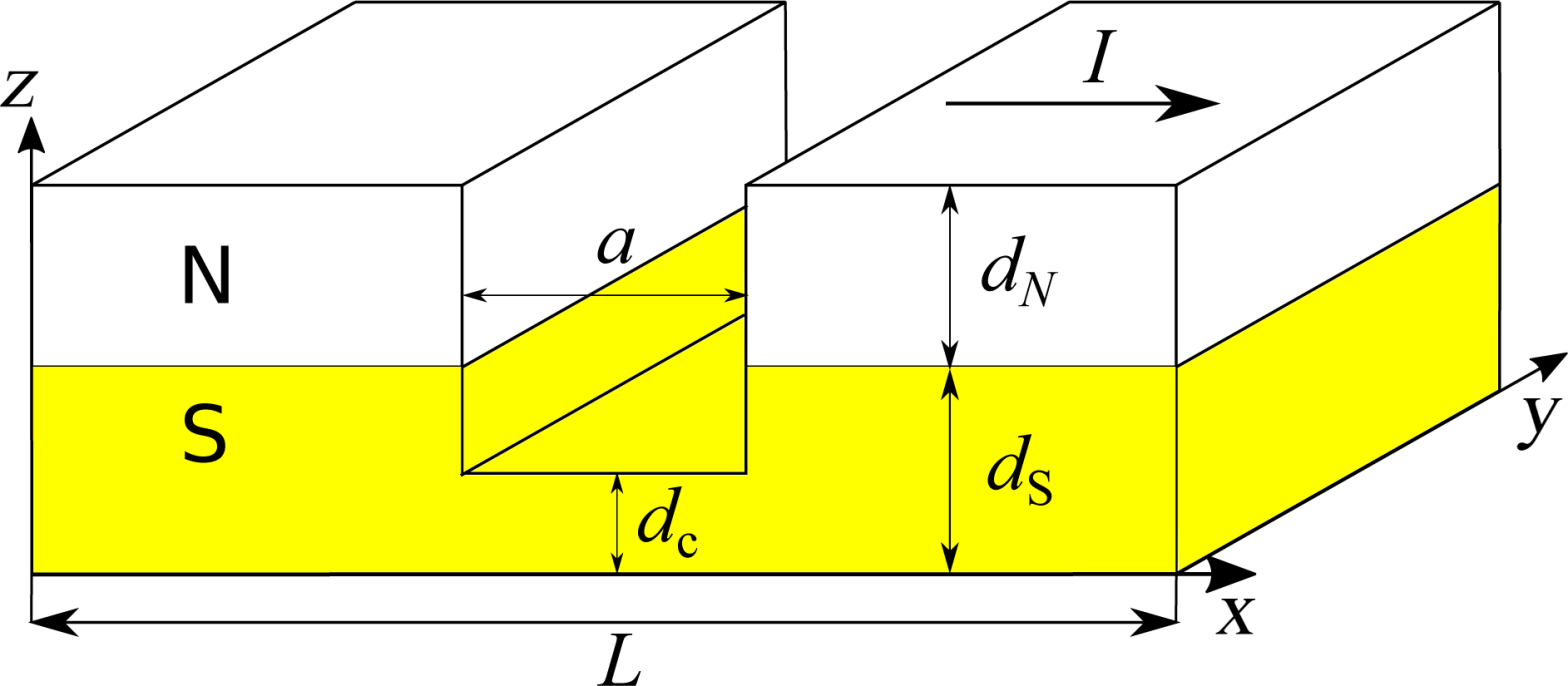
Sketch of a SN-S-SN Josephson junction based on a SN strip of variable thickness.

The model system consists of a SN bilayer strip with length *L* made of a superconducting film with thickness *d*_S_ and a normal metal film with thickness *d*_N_. At the center of the bilayer there is a constriction with length *a* and thickness *d*_c_ where the N layer and, partially, the S layer are removed (Figure 1).

We assume that in our system the current flows in the *x* direction, and in the *y* direction the system is uniform. To find the current–phase relation of such a SN-S-SN Josephson junction at all temperatures below *T*_c_ we solve a 2D Usadel equation for quasiclassical normal *g* and anomalous *f* Green’s functions. With the angle parametrization *g* = cos Θ and *f* = sin Θ exp(iϕ), the 2D Usadel equation in different layers can be written as

[1]
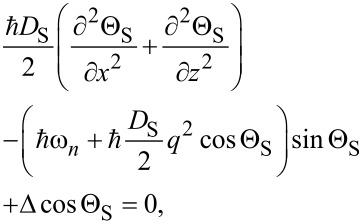


[2]
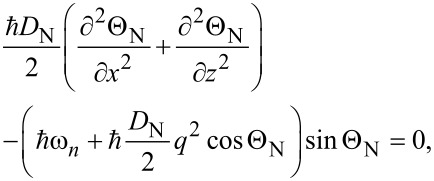


where the subscripts S and N refer to the superconducting and the normal layer, respectively. Here ℏω*_n_* = π*k*_B_*T*(2*n* + 1) are the Matsubara frequencies (*n* is an integer number), **q** = ∇ϕ = (*q_x_*, *q_z_*) is the quantity that is proportional to the supervelocity **v***_s_*, and ϕ is the phase of the superconducting order parameter. Δ is the magnitude of the order parameter, which should satisfy the self-consistency equation

[3]
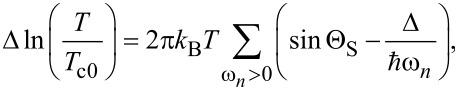


where *T*_c0_ is the critical temperature of the single S layer. We assume that Δ is nonzero only in the S layer because of the absence of attractive phonon-mediated electron–electron coupling in the N layer. Equation 1 and Equation 2 are supplemented by the Kupriyanov–Lukichev boundary conditions [16] between the layers:

[4]
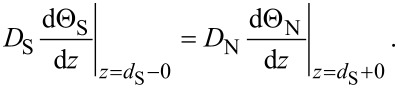


In the model we assume a transparent interface between the N and the S layer, which leads to the continuity of Θ at the NS boundary. At the boundaries of the system with the vacuum we use dΘ/d*n* = 0.

To find the phase distribution ϕ Equation 1–Equation 3 are supplemented by a 2D equation,

[5]
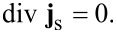


Here, **j**_s_ is the superconducting current density, which is determined by the following expression:

[6]
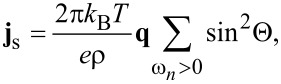


where ρ is the residual resistivity of the corresponding layer. At the SN-interface we use a boundary condition similar to Equation 4, and for the interfaces with the vacuum we use dϕ/d*n* = 0. At the system ends rigid boundary conditions are imposed:

[7]
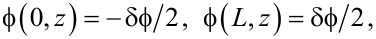


where δϕ is the fixed phase difference between the system ends. This is different from the phase drop near the junction, which we define as

[8]
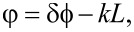


where *k* = *q_x_* (*x* = 0) is far from the constriction (in a similar way φ is defined in [17–18]). The value of *k* is found from the self-consistings solution of Equation 1–Equation 3 and Equation 5.

In numerical calculations we use dimensionless units. The magnitude of the order parameter is normalized by *k*_B_*T*_c0_ = Δ(0)/1.76, lengths are in units of 
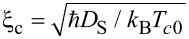
 ≈ 1.33ξ(0), where 
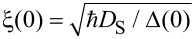
 is the superconducting coherence length at *T* = 0, and the current is in units of the depairing current *I*_dep_ of the superconductor at *T* = 0.

To calculate the CPR we numerically solve Equation 1–Equation 3 and Equation 5 by using an iteration procedure with fixed δϕ. When self-consistency is achieved (we stop the calculations when the maximal relative change of Δ between consequent iterations is less than 10^−4^) the Green’s functions are used to calculate *j*_s_ and the supercurrent per unit of width, *I*_s_:

[9]
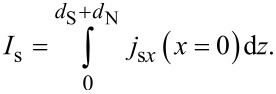


We also compare the calculated CPR with the CPR of a 1D S’-S-S’ system with a large ratio between the diffusion coefficients *D*_S′_/*D*_S_ ≫ 1 (the length of the superconductor S is equal to *a*). For these calculations we use a 1D Usadel equation.

## Results

### Current–phase relation of the SN-S-SN Josephson junction

**Figure 2:**
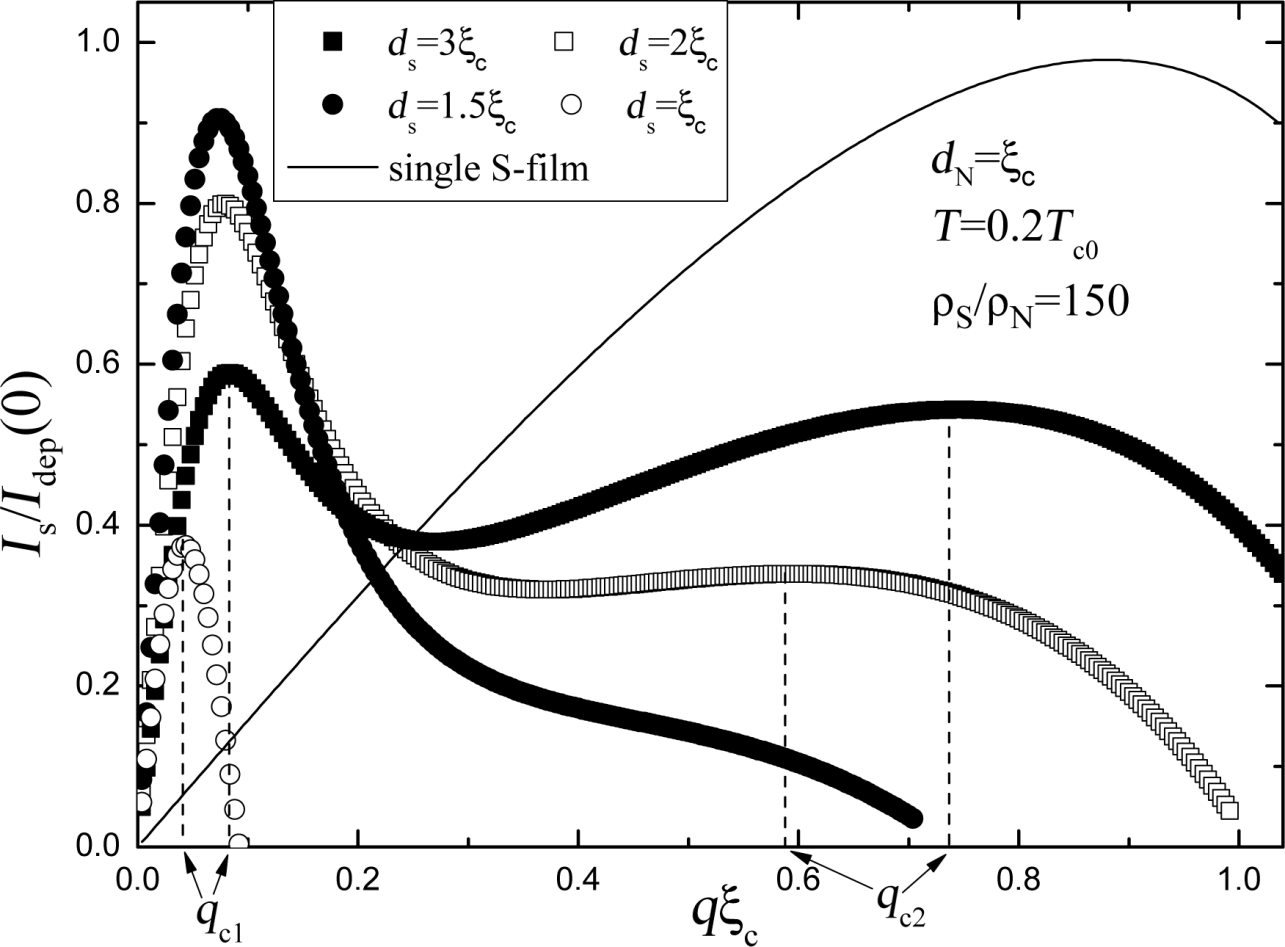
Dependence of the superconducting current *I*_s_ flowing along the SN bilayer on *q* for different *d*_S_. The solid line shows the dependence of *I*_s_ on *q* for the single S strip. The dashed lines show the critical values of *q*. The current is normalized by the depairing current *I*_dep_ of the single S strip with thickness *d*_S_ at *T* = 0.

**Figure 3:**
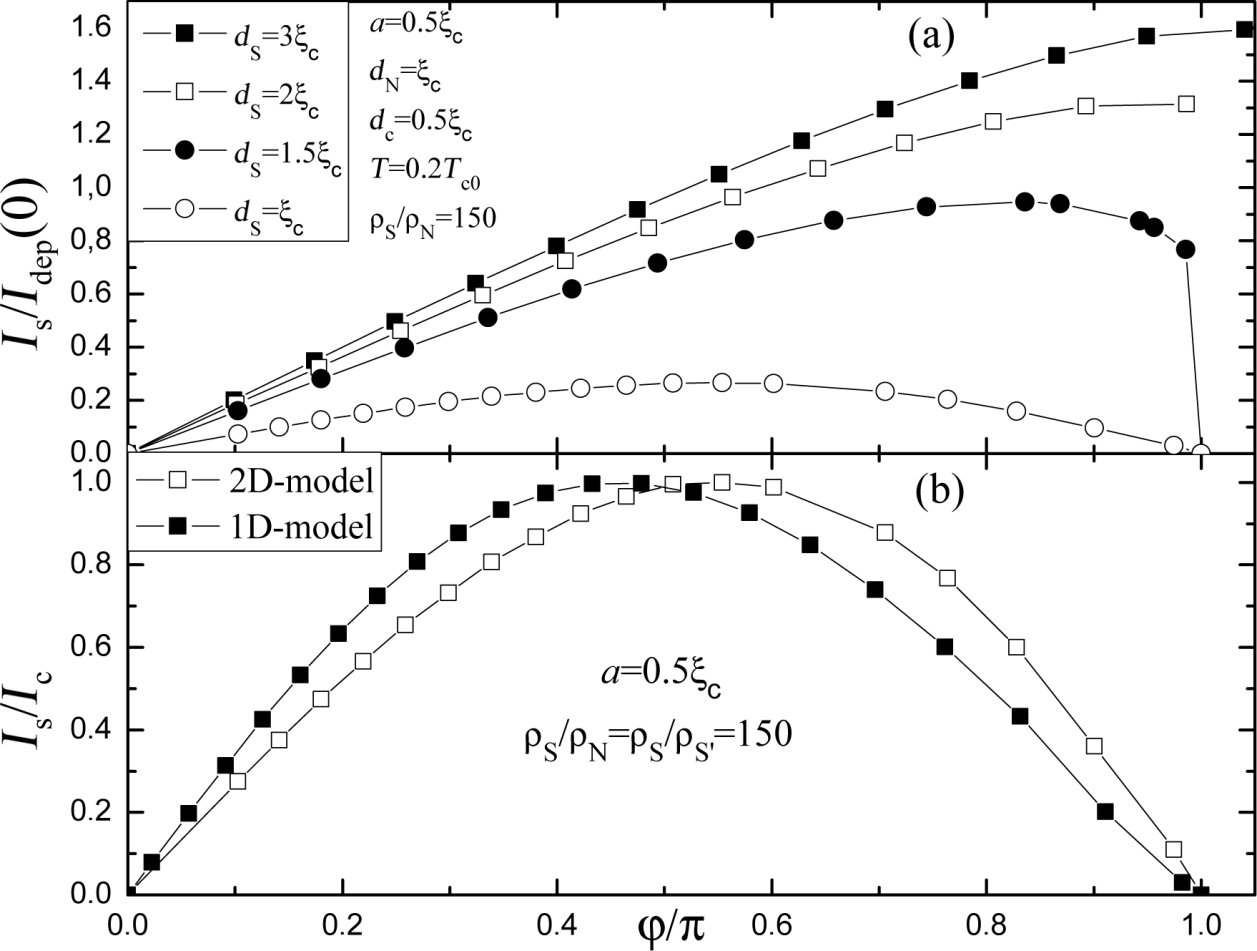
(a) Current–phase relation of a SN-S-SN Josephson junction at different *d*_S_. The current is normalized by the depairing current *I*_dep_ of the single S strip with thickness *d*_c_ at *T* = 0. The junction parameters are shown in the figure. (b) Comparison of current–phase relations calculated on the basis of 1D and 2D models. For the 2D case the parameters are *d*_S_ = *d*_N_ = ξ_c_, *d*_c_ = 0.5ξ_c_, *T* = 0.2*T*_c0_. For the 1D case the temperature *T* = 0.6*T*_c0_ corresponds to *T* = 0.6*T*_c_, where *T*_c_ = 0.32*T*_c0_ is the critical temperature of the SN bilayer with the chosen parameters. The superconducting current is normalized by the critical current of the Josephson junction.

The function *I*_s_(*q*) in the SN bilayer may have one or two maxima depending on the value of *d*_S_ (see Figure 2) or of *d*_N_ (see Figure 3a in [15]). The maximum at small *q* is connected with the suppression of proximity-induced superconductivity in the N layer at 
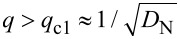
 while the second maximum at 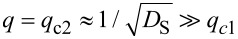 comes from the suppression of superconductivity in the S layer when *q > q*_c2_. The large difference between *q*_c1_ and *q*_c2_ leads to a larger phase concentration in the S constriction (see Figure 1) in comparison with the variable-thickness strip (or Dayem bridge) made of the same material and having the similar geometrical parameters. Because of that, for relatively thin S layers the CPR is single-valued (see Figure 3a), which is not easy to achieve in a Dayem bridge [19]. For relatively large *d*_S_ there is a noticeable contribution to the total supercurrent from the S layer, which means a smaller current (phase) concentration in the constriction like in a common Dayem bridge, and the CPR becomes multi-valued (see Figure 3a for *d*_S_ = 2ξ_c_ and 3ξ_c_).

In some respect, the studied Josephson junction resembles Josephson junctions based on a S’-S-S’system composed of two superconductors S and S’ having *D*_S′_ ≫ *D*_S_ and the same thicknesses *d*_S_ = *d*_S′_ [17,20–21]. A Josephson junction based on this quasi 1D system has a single-valued CPR, which approaches a sinusoidal shape with increasing temperature. In Figure 3b we compare the CPRs calculated for 1D S’-S-S’ and 2D SN-S-SN systems. Since in the 1D model there is no suppression of *T*_c_ through the N layer, we use in the calculations the ratio *T*/*T*_c0_, which corresponds to the ratio *T*/*T*_c_ of the 2D SN structure. Visible differences between the calculated CPRs using different models could be related with a transversal inhomogeneity near the S constriction in the 2D case.

**Figure 4:**
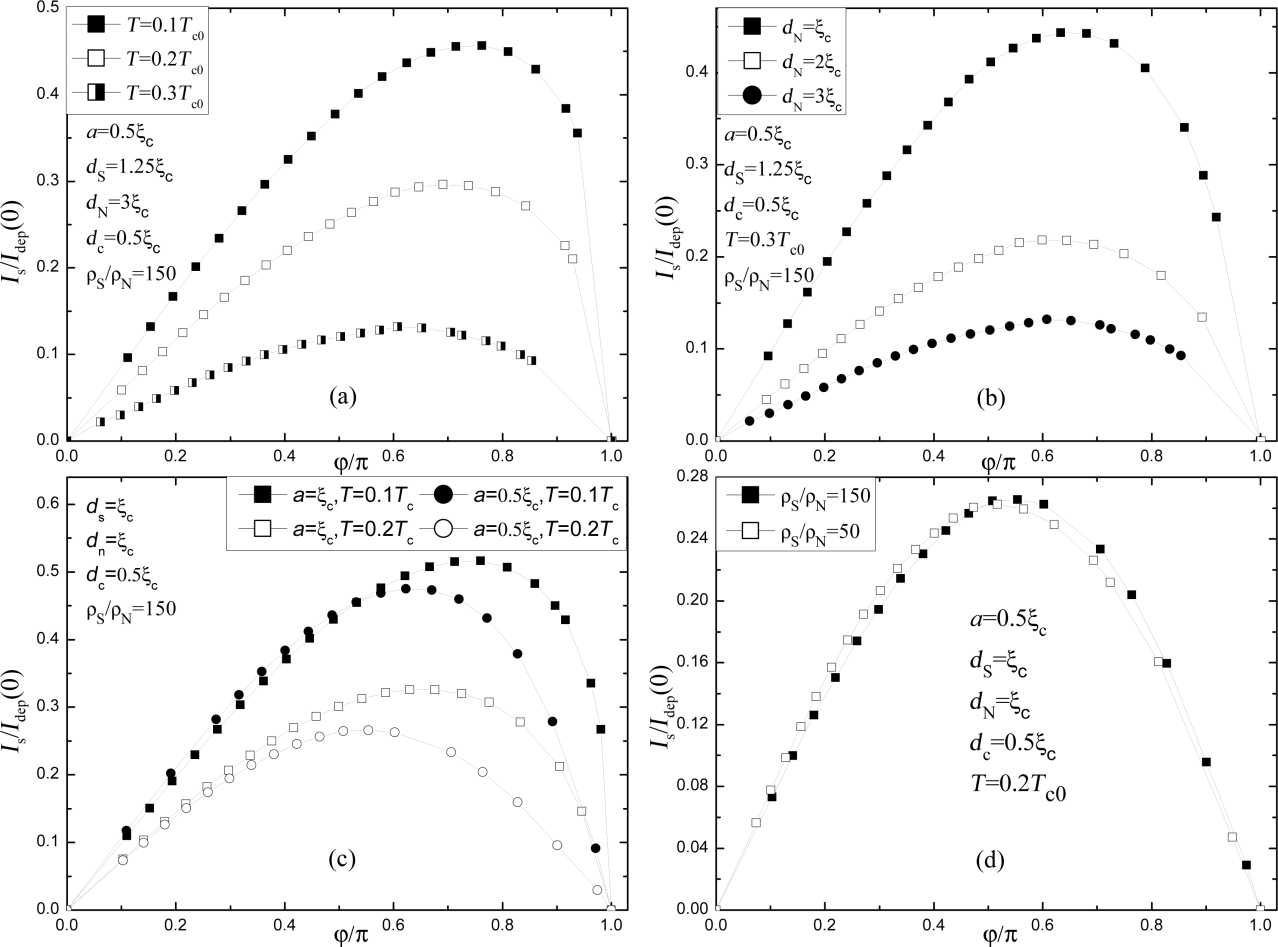
Variation of current–phase relation of SN-S-SN junction as a function of: (a) the temperature; (b) the thickness of the N layer *d*_N_; (c) the length of the constriction, *a*; (d) the ratio between the resistivities. The current is normalized by the depairing current *I*_dep_ of the superconducting strip with thickness *d*_c_ at *T* = 0.

We have also studied the evolution of the CPR of the SN-S-SN Josephson junction when varying different parameters. In Figure 4a we demonstrate that with increase of the temperature the current–phase relation comes close to a sinusoidal shape. At *T* = 0.3*T*_c0_ the amplitude of the first harmonic, sin ϕ, is 0.98*I*_c_ and the amplitude of the second harmonic, sin 2ϕ, is −0.19*I*_c_). This is typical for S’-S-S’ junctions [21] and is related to the increase of the temperature-dependent coherence length ξ(*T*). The effect of different *d*_N_ is shown in Figure 4b. An increase in *d*_N_ leads to a slight shift of the maximum of *I*_s_(φ) to the left and a decrease of *I*_c_. This can be explained by a lowering of *T*_c_ of the SN bilayer for thicker N layers. A lower *I*_c_ means smaller values of *I*_c_*R*_n_. However, as we discuss below, a large value of *d*_N_ provides better cooling of the S constriction and nonhysteretic IVCs.

An increase of the length of the weak link, *a*, leads to the shift of the maximum of *I*_s_(φ) to the right (see Figure 4c) as it is typical for common Josephson junctions with variable thickness. Interestingly, in contrast to common junctions, *I*_c_ increases in the SN-S-SN system. This can be explained by a lower value of the superconducting order parameter in SN banks in comparison with Δ in the S constriction at *I*_s_ = 0. With increasing *a* the superconducting order parameter in the constriction increases and *I*_c_ increases too.

Finally, Figure 4d illustrates that a decrease of the ratio ρ_S_/ρ_N_ to a third of the initial value hardly changes the current–phase relation. Both the critical current and the shape of the CPR vary only little.

### Effect of Joule heating in SN-S-SN junctions

The absence of hysteresis in the current–voltage characteristics is important for devices based on Josephson junctions. The hysteresis in Dayem bridge, variable-thickness, S’-S-S’ or S-N-S junctions is mainly caused by the temperature rise in the weak-link region in the resistive state due to Joule heating and the formation of hot spots [7–9]. A relatively large gap Δ in superconducting banks plays an important role here because it prohibits heat dissipation from the S or the N link at low temperatures *k*_B_*T <* Δ and it leads to hysteresis even for S-N-S junctions of variable thickness [22]. This problem could be resolved by adding heat sinks (voltage leads attached to the N link could play such a role [23]). However, this complicates the geometry of the junction. Local heat production is expected to be large in a SN-S-SN junction due to large critical current density, which is about the depairing current density of the superconductor. As we show below, the presence of a relatively thick N layer with large diffusion coefficient and small minigap in the electron spectra provides efficient cooling of the constriction.

To estimate the increase of temperature in the resistive state we use a two-temperature (2T) model [24–25] for the SN-S-SN junction. We suppose that electron temperature *T*_e_ = *T* + δ*T*_e_ and phonon temperature *T*_p_ = *T* + δ*T*_p_ are close to the substrate temperature, δ*T*_e_, δ*T*_p_ ≪ *T* and do not vary along the thickness. In the N layer the proximity-induced gap (minigap) is small, and, due to the inverse proximity effect, the gap in the relatively thin S layer (*d*_S_ ≤ 1.5ξ_c_) is also suppressed in comparison with a single S layer, which permits heat diffusion from the N to the S layer in SN banks. In the S constriction being in the resistive state at *I > I*_c_ the superconducting order parameter is also suppressed. It allows us to use normal-state heat conductivity both in the SN and the S region in the heat conductance equation for the calculation of δ*T*_e_. This is in contrast to S-N-S and S’-S-S’ junctions where heat conductivity is suppressed in the superconducting banks. In our model Joule dissipation is taken into account only in the S constriction, because in the SN bilayer it is considerably lower due to the much lower resistivity and lower current density. Because of the small length of the constriction and the large difference in diffusion coefficients and thicknesses in constriction and banks we can neglect heat flow to phonons and substrate in the constriction (the main cooling of the junction comes from the diffusion of hot electrons to SN banks). In the SN bilayer *D*_N_ ≫ *D*_S_ and heat diffusion occurs mainly along the N layer. With above assumptions we obtain the following equation for δ*T*_e_:

[10]
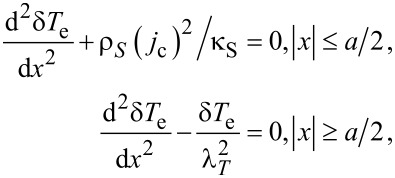


where 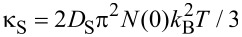 is the electron heat conductivity of the S layer in the normal state, and *N*(0) is the one-spin density of states on the Fermi level,

[11]
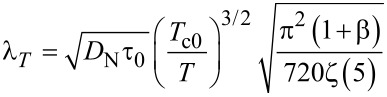


is the thermal healing length, β = [γτ_esc_ 450ζ(5)*T*/[τ_0_π^4^*T*_c0_], ζ(5) ≈ 1.03, τ_esc_ is the escape time of nonequilibrium phonons to the substrate, γ = 8π^2^C_e_(*T*_c0_)/C_p_(*T*_c0_) is the ratio between electron and phonon heat capacity at *T* = *T*_c0_ and τ_0_ determines the strength of electron–phonon inelastic scattering in the S and the N layer (see Equations 4 and 6 in [25]). For τ_0_ we use the smallest time for S and N materials due to the assumably good transfer of electrons between the S and the N layer and the small thickness of the layers. On the boundary between S and SN regions we use a continuity of the electron temperature, δ*T*_e_|*_a/2−0_* = δ*T*_e_|*_a/2+0_*, and of the heat flux


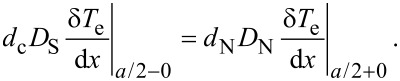


Using Equation 10 and above boundary conditions, we find the maximal temperature increase in the constriction:

[12]
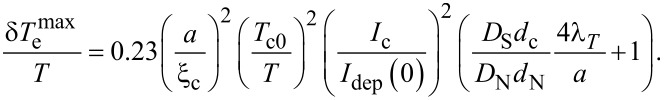


In the following estimations we use the parameters of NbN (S layer) and Cu (N layer): *T*_c0_ = 10 K, *D*_S_ = 0.5 cm^2^/s, ρ_S_ = 200 μΩ·cm, *D*_N_ = 40 cm^2^/s, ρ_N_ = 2 μΩ·cm, τ_0_ = 1 ns (theoretical estimation for NbN taken from [25]), ξ_c_ = 6.4 nm, γ = 9, *d*_S_ = 1.25ξ_c_, *d_N_* = 2ξ_c_, τ_esc_ = 4(*d*_N_ + *d*_S_)/*u* ≈ 41 ps (*u* = 2·10^5^ cm^2^/s is the mean speed of sound), *T*/*T*_c0_ = 0.3, *T*_c_/*T*_c0_ = 0.43, *a*= 0.5ξ_c_, *d*_c_ = 0.5ξ_c_. With these parameters β ≈ 0.53, *I*_c_ ≈ 0.22*I*_dep_(0) (see Figure 4b) and 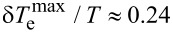 is small, thanks to *D*_N_ ≫ *D*_S_ and *d*_N_ ≫ *d*_c_.

## Discussion

We use an Usadel model to calculate the current–phase relation of a SN-S-SN Josephson junction based on a high-resistivity superconductor and a low-resistivity normal metal. In [15], from comparison of experiment and theory it was concluded that the Usadel model underestimates proximity-induced superconductivity in the N layer and overestimates the inverse proximity effect in the S layer in NbN/Al, NbN/Ag and MoN/Ag bilayers. Namely, the suppression of the critical temperature of the SN bilayer is smaller while the change in magnetic field penetration depth of the SN bilayer is larger than the Usadel model predicts. Therefore, the present results should be considered only as a route for a possible experimental realization of SN-S-SN Josephson junctions. They demonstrate that the thickness of the S layer should not exceed ca. 1.5ξ_c_, otherwise the current–phase relation is not single-valued for reasonable lengths and thicknesses of the S constriction. The thickness of the N layer should not be too small (a small *d*_N_ leads to large overheating) and not too large (a larger *d*_N_ leads to lower *T*_c_ and smaller *I*_c_ at a fixed substrate temperature).

Our results show that the SN-S-SN Josephson junction in many respects resembles Dayem bridge, variable-thickness, S’-S-S’ or S-N-S junctions. The product

[13]
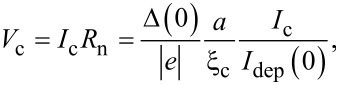


can reach 0.5Δ(0)/|e| at a low temperature (*T* = 0.1*T*_c0_) and *a* = ξ_c_ (see Figure 4c) due to use of a superconductor in the constriction area, instead of a normal metal as in [13]. In case of NbN with *T*_c0_ = 10 K one may have *V*_c_ = 0.75 mV but according to Equation 12), 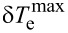 will be larger than *T* when using these parameters. However there is the hope, that the critical temperature of a real SN bilayer is higher than the Usadel model predicts (see discussion above) and therefore large *I*_c_ values could be reached at higher operating temperatures *T*/*T*_c0_, leading to a drastic reduction of 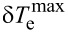 (see Equation 12).

SN-S-SN junctions made of a NbN/Al bilayer have been fabricated recently [26] and indications of the Josephson effect (the presence of Shapiro steps and a Fraunhofer-like dependence of the critical current on the magnetic field) have been observed. But due to not optimized parameters (*d*_S_ = *d*_c_ ≈ 15 nm ≈ 2.3 ξ_c_, *d*_N_ ≈ 29 nm ≈ 4.5 ξ_c_, *a* = 20 nm ≈ 3.1 ξ_c_) the IV curves were hysteretic already at temperatures close to the critical temperature and the width of Shapiro steps did not follow the theoretical expectations [26]. Modern technology allows one to fabricate constrictions with lengths of about 5 nm, which is smaller than ξ_c_ in NbN, with the help of helium ion beam lithography. The successful implementation of this method could lead to the creation of low-temperature nanoscale Josephson junctions and arrays of them. For example, SN-S-SN junctions can be promising to use in programmable voltage standards [1], where a large value of *V*_c_ allows for a reduction of the number of junctions and for the use of Shapiro steps of orders higher than one. Nonhysteretic current–voltage characteristics with large *V*_c_ at low temperatures allow for the use of these structures for various low-temperature applications, e.g., particle detectors [3].

## Conclusion

We have calculated the current–phase relation of a Josephson junction based on a SN-S-SN strip of variable thickness, where S is a dirty superconductor with large normal-state resistivity and N is a low-resistivity normal metal. We find a range of parameters for which the CPR is single-valued, is close to a sinusoidal shape, and *I*_c_*R*_n_ ≤Δ(0)/2|e|. Our estimations demonstrate that a relatively thick N layer serves as effective heat conductor yielding weak overheating and a nonhysteretic current–voltage characteristic of the SN-S-SN Josephson junction.
